# Supplementary material for: Polyethylene glycol precipitation is an efficient method to obtain extracellular vesicle-depleted fetal bovine serum
Source: PLoS One. 2023 Dec 5;18(12):e0295076. doi: 10.1371/journal.pone.0295076 (PMC10697576; doi:10.1371/journal.pone.0295076)
Supplement: S1 Table — (DOCX) [file pone.0295076.s001.docx]

| Gene | Forward | Reverse |
| --- | --- | --- |
| GAPDH | ATCTTCTTTTGCGTCGCCAG | TTCCCCATGGTGTCTGAGC |
| COL2A1 | CACGTACACTGCCCTGAAGGA | CGATAACAGTCTTGCCCCACTT |
| CCND1 | ATCAAGTGTGACCCGGACTG | CTTGGGGTCCATGTTCTGCT |
| SIRT1 | AGAGCCTCACATGCAAGCTCTAG | GCCAATCATAAGATGTTGCTGAAC |
| COL1A1 | AGATCGAGAACATCCGGAG | AGATCGAGAACATCCGGAG |
| TGFb1 | GAGGTCACCCGCGTGCTA | TGCTTGAACTTGTCATAGATTTCGTT |
| TIMP1 | CAATTCCGACCTCGTCATCAG | TATACATCTTGGTCATCTTGATCTCATAAC |

Sequence of primers for real-time PCR analysis.
