# Supplementary figures and images for: Polyethylene glycol precipitation is an efficient method to obtain extracellular vesicle-depleted fetal bovine serum
Source: PLoS One. 2023 Dec 5;18(12):e0295076. doi: 10.1371/journal.pone.0295076 (PMC10697576; doi:10.1371/journal.pone.0295076)

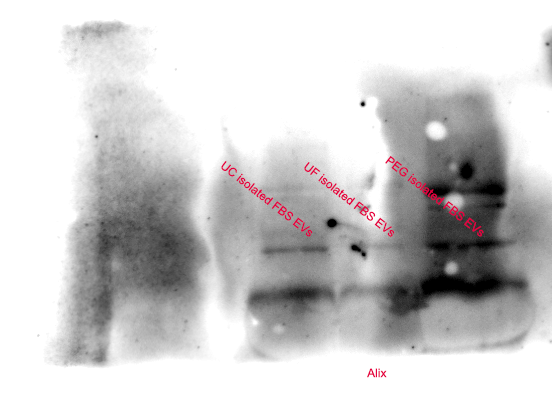

Supplement: S1 File — (ZIP) [file pone.0295076.s002.zip › Alix.tif]

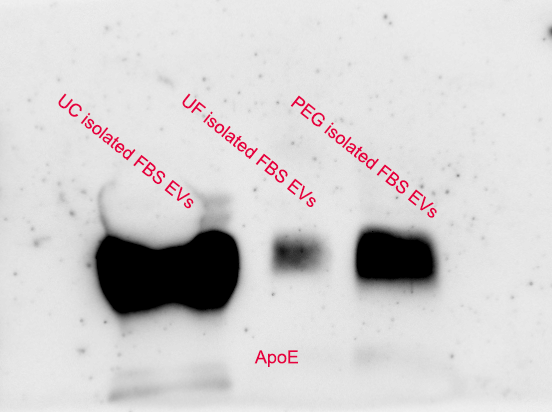

Supplement: S1 File — (ZIP) [file pone.0295076.s002.zip › ApoE.tif]

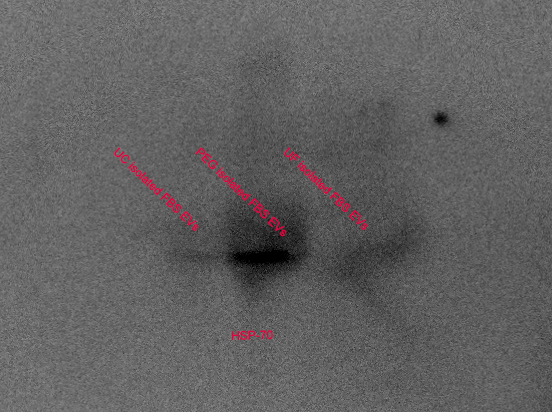

Supplement: S1 File — (ZIP) [file pone.0295076.s002.zip › HSP-70.tif]
