# Supplementary material for: Polyethylene glycol precipitation is an efficient method to obtain extracellular vesicle-depleted fetal bovine serum
Source: PLoS One. 2023 Dec 5;18(12):e0295076. doi: 10.1371/journal.pone.0295076 (PMC10697576; doi:10.1371/journal.pone.0295076)
Supplement: Attachment — Submitted filename: Rebuttal Letter.docx [file pone.0295076.s003.docx]

1. Protein content cannot be a criterion to measure the purity of exosomes according to the MISEV guidelines. We suggest that authors adhere to MISEV guidelines for characterisation.
   a. Protein should be complemented with CD83or CD81 or CD9.

Response: CD81 was detected in the three isolated FBS EVs, together with HSP-70. Now one more EV marker Alix has been supplemented.

b. Lipid concentrations of the exosomes should be included, and thus P/L ration will be available, which is an indirect measure of Exosome preparation or depletion.

Response: EVs are a subgroup of nanosized particle enclosed by a lipid bilayer membrane. The lipid concentration couldn’t be an indirect measure of EVs depletion. To test EV preparation, “NTA+TEM image + protein marker” is the gold principle. But we tested the one lipoprotein marker ApoE due to the overlap in density between high-density lipoprotein and EVs. The image band showed more ApoE were isolated with FBS EVs by performing UC, compared with by performing PEG precipitation. In addition, though the much essence loss happened by performing UF due to its low selectivity, the faint band of ApoE was observed in UF isolated FBS EVs, meaning some high-density lipoproteins (molecule weight below 100 kDa) without aggregation could be retained in UF EVdFBS.

c. Functional assay for Exosome purity like acetylcholine esterase should be used.

Response: In terms of EVs characterization, the acetylcholine esterase is not a common marker to be used to determine the EVs purity based on the paper: *Acetylcholinesterase is not a generic marker of extracellular vesicles*. On the other hand, in cell culture with serum, most acetylcholinesterase is from the serum, meaning acetylcholinesterase interferes the content of FBS EVs, which make the comparison of acetylcholinesterase activity among three isolated FBS EVs meaningless. Therefore, the acetylcholine esterase is not necessary to be observed.

2. Fig 1,2,3 is monotonous and repetitive. It can be combined and compared
   a. Workflow can be one figure, and B, C, and D of Figures 1,2, and 3 can be a single figure with comparisons for easy understanding to the readers.

Response: The figure now is shown below:

3. The authors have directly used PEG 6K. They should have performed a preliminary experiment with different molecular weights (PEG; 3,4,5,6,10 etc) and then an ideal PEG should be used for further experimentation.

Response: We chose **PEG 6K** based on the previous study and an abundant of relevant literature. (*ExtraPEG: A Polyethylene Glycol-Based Method for Enrichment of Extracellular Vesicles; Low concentration of polyethylene glycol facilitates separation of extracellular vesicles from bronchoalveolar lavage fluid*). For example, the paper: *precipitation with polyethylene glycol followed by washing and pelleting by ultracentrifugation enriches extracellular vesicles from tissue culture supernatants in small and large scales*, exhibited that PEG was a reliable method to enrich sEVs from cell culture supernatants, and PEG 6K play a more effective role in sEVs precipitation, compared with PEG 8K and PEG 20K.

4. For biophysical characterization along with NTA, AFM of TEM should be incorporated as per MISEV guidelines.

Response: The biophysical characterization of PEG isolated FBS EVs now has been observed through TEM.

5. For EV profiling, at least three markers should be analysed (MISEV guidelines; represented blots are of inferior quality and need to be replaced.

Response: Now one more EV marker Alix has been supplemented, together with ApoE mentioned in comment 1b.

6. EV-depleted FBS mass spec may be performed and should be extensively characterised according to the work's rationale. The following experiments would add value to the paper
   a. Exosome depletion can be verified by NTA/DLS.

Response: The EVs depletion could be seen in Fig 1, 2. Summarily, FBS EV depletion was comparable between PEG (95.6%) and UF (96.6%) but less by UC (82%), as compared to FBS.

b. Exosome depletion can be verified by fluorescent dyes like bodipy etc.

Response: Fluorescent dyes is not necessary. Fluorescence dyes has been widely used for EV tracking to studied EV uptake by target cells and their in vivo distribution.

1. To make EVdFBS, normally several milliliter FBS are needed, therefore it will cost a lot of dyes if dyes are directly added into FBS and researches evaluate EVs depletion through determining the fluorescence in corresponding EVdFBS. 2. It is not feasible to determining the isolated FBS EVs through labeling isolated FBS EVs. Because the isolated FBS EVs is used to indirectly evaluate the EVs depletion, therefore the proportion of isolated FBS EVs (the purity of isolated FBS EVs) in isolated materials is supposed to be determined. Therefore, the EVs characterization is needed. The non-EV nanoparticles and dyes aggregation would be regarded as labeling isolated FBS EVs in fluorescence image.

c. Different FBS should be run on an SDS PAGE to see the proteins that are depleted

Response: We did test the total protein concentration of FBS and other four EVdFBS through microBCA in Fig 2B.

7. Figure legends and labels should be rechecked for grammatical errors.

Response: They has been rechecked.

8. Growth factor markers can be analysed to support the cell growth data.

Response: Some genes important for cell proliferation (i.e., CCND1, SIRT1) have been test in hADSCs, which are cultured in different EVdFBS media, and the image is shown below.

9. mRNA levels of Col1a1 and TIMP1 cannot satisfy the analysis of collagen metabolism. Thus, additional experiments need to be incorporated.

Response: Based on the previous study, for example, the papers: *Mechanical strain affects collagen metabolism-related gene expression in scleral fibroblasts*; *Exosomes secreted by adipose-derived mesenchymal stem cells regulate type I collagen metabolism in fibroblasts from women with stress urinary incontinence*. In these papers, the mRNA levels of COL1a1, TIMP1 in vaginal fibroblasts/ scleral fibroblasts were determined to evaluate the collagen metabolism.

In our study, the EVs derived from ADSC-PEG EVdFBS media still retained bioactive, therefore their effect on collagen metabolism was selected as an example and Col1a1, TGFb1, TIMP1 were markers of human knee fibroblasts, because the Col1a1 is the most abundant [collagen](https://en.wikipedia.org/wiki/Collagen) of the human body. It forms large, eosinophilic fibers known as collagen fibers. The TGFb1 appears in high concentrations in fibrotic cardiac tissue and is a potent inductor of tissue collagen deposition. It is accepted that TGF-beta 1 is a potent stimulator of collagen secretion by fibroblasts. The TIMP1 is a [glycoprotein](https://en.wikipedia.org/wiki/Glycoprotein) and a natural [inhibitor](https://en.wikipedia.org/wiki/Enzyme_inhibitor) of the [matrix metalloproteinases](https://en.wikipedia.org/wiki/Matrix_metalloproteinase) (MMPs), a group of peptidases involved in degradation of the [extracellular matrix](https://en.wikipedia.org/wiki/Extracellular_matrix). Therefore, the mRNA level of Col1a1 and TIMP1 could be used to evaluate the collagen metabolism, specifically collagen I metabolism.

10. Authors should refer to and discuss the following paper: Cost-effective Strategies for Depletion of Endogenous Extracellular Vesicles from Fetal Bovine Serum.

Response: The authors also found the effective particles could be removd from FBS through PEG 4K precipitation. The final PEG solution is 3.2%, however, in our study the particles in PEG EVdFBS have increased following the decrease of final PEG solution, we speculated different commercial FBS caused this. In terms of the status of cells cultured in PEG EVdFBS, the primary adipose tissue derived cells cultured in 10% PEG 4000 mediated EVdFBS expressed CD44 (99.7%), CD105 (99.8%) and CD29 (97.2%), as markers for stem cells in this study. While we focused on the differentiation capacity of cells, which was not influenced. Moreover, in our study the cell-derived EVs obtained from PEG EVdFBS supernatant were obtained and still retained bioactive, meaning the PEG EVdFBS supports cell-EVs enrichment.

11. Chemicals and reagents procurement details (catalogue numbers) should be mentioned.

Response: Yes, they are rechecked.
